# Announcement

**Published:** 1899-04

**Authors:** 


### *EDITORIAL NOTES AND COMMENTS.*

The Business office of THE JOURNAL-RECORD is 305, 306 Fitten Building.

The Editorial office is Rooms 401, 402 Grand Opera House.

Address all Business communications to Dr. M. B. Hutchins, Mgr.

Make remittances payable to THE ATLANTA JOURNAL-RECORD OF MEDICINE.

On matters pertaining to the Editorial and Original Communications address Dr. Dunbar Roy, Grand Opera House, Atlanta.

Reprints of original articles will be furnished at cost price. Requests for the same should always be made *on the manuscript.*

We will present, post-paid, on request, to each contributor of an original article, twenty (20) marked copies of THE JOURNAL-RECORD containing such article.

### ANNOUNCEMENT.

THE ATLANTA MEDICAL AND SURGICAL JOURNAL and THE SOUTHERN MEDICAL RECORD have consolidated to form THE ATLANTA JOURNAL–RECORD OF MEDICINE, of which this is the first issue. The management of the new publication desires it understood that the combination is in no wise an absorption of one journal by the other. It is a union of interests, as it was deemed expedient, in consideration of the unnecessarily large number of medical journals already in the field, to compress the two local journals into one.

All existing contracts of whatever nature will be carried out, despite the radical change.

It is our desire to make THE ATLANTA JOURNAL–RECORD OF MEDICINE the chief exponent of medicine in the South, and to this laudable end we invite the cooperation of our friends.

